# Applications and Prospects of Metabolomics and Lipidomics Technologies in the Study of Livestock and Poultry Meat and Egg Quality

**DOI:** 10.3390/foods15081401

**Published:** 2026-04-17

**Authors:** Keyu Li, Ying Lu, Dan Yue, Yuwei Qian, Huaijing Liu, Zhengmei Sheng, Jinpeng Shi, Yang Yang, Jiao Wu, Dongmei Xi, Yuqing Chong

**Affiliations:** Yunnan Provincial Key Laboratory of Animal Nutrition and Feed, Faculty of Animal Science and Technology, Yunnan Agricultural University, Kunming 650201, China; 17787916184@163.com (K.L.); yinglu_1998@163.com (Y.L.); danyue0528@foxmail.com (D.Y.); 15368050094@163.com (Y.Q.); 18361309553@163.com (H.L.); 18008847283@163.com (Z.S.); sjp020607@163.com (J.S.); 18287913848@163.com (Y.Y.); wujiao@ynau.edu.cn (J.W.)

**Keywords:** lipidomics, metabolomics, meat quality, egg quality, artificial intelligence (AI)

## Abstract

As essential branches of systems biology, metabolomics and lipidomics systematically reveal the composition, dynamic changes, and biological functions of small-molecule metabolites and lipids using high-throughput analytical techniques. This review examines the application of these omics technologies in evaluating livestock and poultry meat and egg quality, focusing on their roles in elucidating the molecular mechanisms behind key traits such as flavor, tenderness, and nutritional value. By identifying key metabolic markers—including glutamic acid, inosine monophosphate, and specific triglycerides—the intrinsic links between these markers and intramuscular fat deposition, flavor precursor formation, and antioxidant capacity are highlighted. Furthermore, this paper emphasizes the transformative impact of integrating multi-omics data with artificial intelligence (AI). AI-driven analytical frameworks are overcoming the limitations of traditional high-dimensional data processing, enabling robust biomarker discovery, predictive modeling for product quality, and reverse design for genetic improvement. Ultimately, the synergistic application of metabolomics, lipidomics, and AI will drive the development of modern animal husbandry toward intelligent, predictable, and precision-based production.

## 1. Introduction

Metabolomics is one of the key omics approaches in animal husbandry research, and its core advantage lies in the ability to systematically analyze small-molecule metabolites not covered by genomics, transcriptomics, and proteomics [1,2]. In addition, when elucidating the response mechanisms of organisms to environmental impacts and changes in biochemical activities, metabolomics can more directly and dynamically reflect the physiological state and regulatory endpoints of biological systems compared with transcriptomics and proteomics, thus playing an irreplaceable role in explaining environmental stress and metabolic regulatory mechanisms [3,4]. Lipidomics is a discipline that systematically studies the composition, structure and function of all lipid molecules in organisms, aiming to conduct qualitative and quantitative analysis of lipids and reveal their molecular mechanisms involved in physiological and pathological processes at the cellular, organ and even whole-life levels [5,6]. As an important branch of metabolomics, lipidomics focuses on the specific category of lipid metabolites [7]. Compared with traditional metabolomics, lipidomics places more emphasis on the functional significance of subtle differences in lipid structures such as carbon chain length, unsaturation and isomers, providing higher-resolution mechanistic information for metabolic regulation research.

In livestock and poultry breeding and production, meat and egg quality is no longer a traditional accessory trait but a core economic trait on par with traditional key indicators such as growth rate and feed conversion rate, directly determining the economic returns of production [8]. Specifically, qualities such as tenderness, flavor, and nutritional composition affect the market value of products, consumers’ willingness to purchase, and the response to healthy consumption demands, thereby directly influencing the income and profits of breeding enterprises. Therefore, they have become core economic factors that must be prioritized and improved in production. Therefore, this review aims to systematically summarize how metabolomics and lipidomics have been applied to elucidate the molecular basis of meat and egg quality traits [9,10,11]. Meat and egg quality traits are key determinants of product value: sensory attributes such as tenderness and flavor directly shape consumer experience and purchasing decisions, while nutritional attributes including fatty acid composition, cholesterol content, and trace element enrichment address the growing demand for health-oriented animal products [12,13,14]. By synthesizing current evidence, we identify robust biomarkers and critically assess methodological challenges, with the ultimate goal of providing a roadmap for future research that moves from descriptive correlation to mechanism-based validation.

The combination of metabolomics and lipidomics provides a key technical means for clarifying the formation mechanism of meat and egg quality by systematically analyzing the overall profiles of small-molecule metabolites and lipids in organisms. In modern livestock and poultry research, the combined use of metabolomics and lipidomics can elucidate the mechanism of meat and egg quality, identify biomarkers such as phosphatidylcholine, clarify their metabolic pathways, and provide a theoretical basis for efficient livestock and poultry breeding, improvement of meat and egg quality, disease diagnosis, and the discovery of meat and egg quality biomarkers.

## 2. Overview of Metabolomics and Lipidomics

As important branches of systems biology, metabolomics and lipidomics profoundly reveal the ultimate phenotypes of life processes by finely depicting the overall profiles of all small-molecule metabolites and lipids in organisms. They act like sophisticated “molecular microscopes”, which can accurately analyze the dynamic changes of flavor precursors, nutritional components and potential biomarkers, and precisely reveal the intrinsic physiological states and molecular regulatory networks of livestock and poultry during growth, disease resistance, reproduction and stress processes, thus having far-reaching significance for improving the production efficiency and product quality of animal husbandry.

### 2.1. Overview of Metabolomics

Metabolomics is an important component of systems biology, which aims to conduct qualitative and quantitative analysis of all small-molecule metabolites with a molecular weight of <1500 Da in biological systems [15,16]. Metabolomics is a research method that conducts qualitative and quantitative analysis of metabolites through mass spectrometry, chromatography and nuclear magnetic resonance technology, and combines bioinformatics to explore their biological significance [17,18,19]. By revealing the variation rules of metabolites in biological systems under specific physiological, pathological or environmental interventions, it elucidates the relationship between metabolic networks and life activities [20,21].

In metabolomics studies, experimental designs typically require at least six biological replicates per group, randomized sample order, and the use of mixed quality control (QC) samples to monitor instrument stability. During sample preparation, liquid nitrogen or cold organic solvents are often used to rapidly inactivate enzymes, and a methanol/chloroform/water system is commonly employed for metabolite extraction [22,23,24,25,26]. The commonly used analytical platforms mainly include gas chromatography–mass spectrometry (GC-MS), liquid chromatography–mass spectrometry (LC-MS), capillary electrophoresis-mass spectrometry (CE-MS) and nuclear magnetic resonance (NMR) technology [27]. Among them, GC-MS is suitable for the analysis of low-molecular-weight (approximately 50–600 Da) volatile compounds, and for highly polar and non-volatile metabolites, chemical derivatization is usually required before analysis [28]. In comparison, LC-MS generally does not require a derivatization step before analysis, has a wide analytical range that can cover a variety of metabolites with different physical and chemical properties, and also has a wide applicable molecular weight range [29]. Although CE-MS is not as sensitive as LC-MS, it has high analytical stability and good reproducibility, and shows significant advantages especially when dealing with scarce biological samples [30]. In addition, the detection sensitivity and resolution of NMR technology are relatively low, and it is usually only suitable for the detection of metabolites in the concentration range of micromolar to millimolar (μM–mM); thus, it is difficult to achieve accurate quantification of low-abundance metabolites. However, NMR technology features simple sample pretreatment, rapid analysis process and excellent analytical reproducibility.

Data acquisition is performed under optimized chromatographic and mass spectrometric conditions, with QC samples inserted regularly throughout the analytical sequence. Bioinformatics processing includes peak picking, retention time alignment, missing value imputation, and normalization, commonly using total ion current normalization or QC-based normalization [31,32,33]. For data processing, a variety of open-source software tools can be used. For example, the XCMS online platform supports the reading of raw data in formats such as mzXML, NetCDF, and mzData, and after preprocessing, it performs metabolite identification, statistical analysis, and pathway enrichment analysis based on the METLIN database [34]. Software such as MetaboAnalyst 5.0 and MS-DIAL can also be used for data preprocessing, normalization, and multivariate statistical analysis [35,36]. Commonly used databases for metabolite identification include HMDB (Human Metabolome Database), METLIN, GMD (Golm Metabolome Database) and MassBank [37,38,39,40]. After identification, further functional annotation and pathway interpretation of differential metabolites can be performed using IMPaLA, MSEA (Metabolite Set Enrichment Analysis), as well as pathway databases such as KEGG, Recon1, and Biocyc, thereby linking differential metabolites to biological interpretation [41,42,43].

### 2.2. Overview of Lipidomics

Lipidomics was first proposed in 2003 and is an important branch of metabolomics [44,45]. It aims to conduct qualitative identification, quantitative analysis and dynamic tracking of all lipid molecules in a cell, tissue, organ or the entire organism of a living being under specific physiological states [46]. Lipidomics is a research method that analyzes lipid molecules through mass spectrometry and chromatography technologies, and combines bioinformatics to determine their biological significance [47]. By revealing the variation rules of lipids in organisms under specific physiological or environmental interventions, it elucidates the lipid metabolic network.

In lipidomics studies, the experimental design is similar to that of metabolomics, requiring at least six biological replicates per group, randomized sample order, and the use of mixed quality control (QC) samples to monitor instrument stability. Sample preparation typically employs methods such as Folch or Bligh-Dyer, using a chloroform/methanol/water system to efficiently extract lipids and minimize degradation [48,49]. Since most lipid molecules are high-polarity and large-molecular-weight compounds, such as triglycerides and phospholipids [44], they are difficult to directly vaporize in GC-MS analysis due to poor thermal stability or low volatility. Therefore, GC-MS is generally not used as a universal analytical tool in lipidomics, but mainly for the specific analysis of certain classes of volatile lipids after pretreatment such as derivatization. In contrast, LC-MS does not require derivatization, has a wide scope of application, and can achieve multi-level research objectives ranging from comprehensive lipidome coverage analysis to targeted and precise detection. In LC-MS analysis, reversed-phase liquid chromatography (RPLC) and hydrophilic interaction liquid chromatography (HILIC) are currently the two most commonly used separation modes [50,51]. In addition, supercritical fluid chromatography–mass spectrometry (SFC-MS) is gradually becoming an important tool in lipidomics research [52,53]. In targeted lipid analysis, the multiple reaction monitoring (MRM) strategy based on triple quadrupole mass spectrometry is widely adopted due to its high sensitivity and high selectivity [54]. For lipidomics data processing, the open-source R/Bioconductor package ‘lipidr’ can be used to perform data mining and analysis on lipidomics datasets [55]. Subsequently, LIPID Maps and CHEBI (Chemical Entities of Biological Interest) can be employed for identification [56,57]. Once identification is complete, databases such as KEGG and VANTED can be used to perform functional annotation and pathway interpretation of differentially expressed lipids, thereby linking these lipids to biological interpretations [41,58]. Additionally, LipidSig 2.0 can be utilized during data processing. LipidSig 2.0 enables direct data preprocessing, lipid ID annotation, differential expression analysis, enrichment analysis, and network analysis, thereby simplifying lipidomics data analysis [59].

### 2.3. Advantages of Their Combined Application

**Figure 1:**
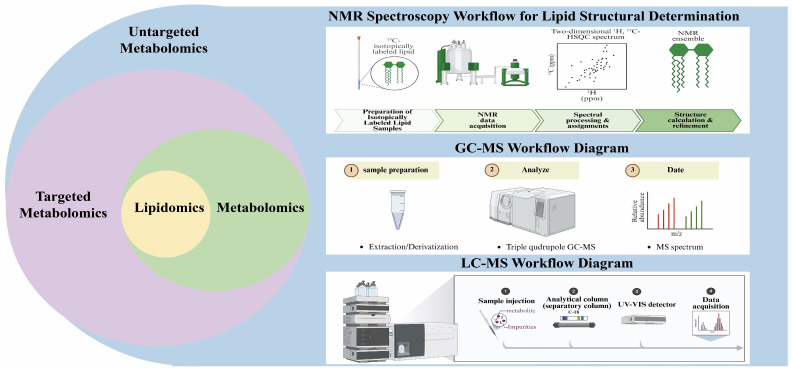
Shows the main technical flowcharts of metabolomics and lipidomics.

Metabolomics focuses on polar metabolites such as sugars, nucleotides, amino acids, and organic acids, which are mostly water-soluble and thus reflects the core metabolic pathways of cells [60,61]. Lipid molecules not only serve as the structural basis of cell membranes but also play important roles as energy stores, signaling molecules, and regulators of gene expression. Due to their hydrophobic nature, the coverage of lipid molecules that can be studied by metabolomics alone is relatively limited. In contrast, lipidomics enables comprehensive identification and quantification of lipid molecules [62,63,64]. In chicken, integrated metabolomic and lipidomic analysis revealed that the increase in intramuscular fat deposition with age is mainly attributed to the accumulation of triglycerides, fatty acids, and cholesteryl esters, and identified key flavor metabolites such as LysoPS 18:1 [65].Therefore, the combined application of metabolomics and lipidomics enables the acquisition of a more complete molecular profile, clarifies the synergistic changes between metabolites and lipids, and reveals their associated mechanisms, thereby facilitating a comprehensive understanding of the formation mechanisms underlying flavor and nutritional quality. The main technical workflows of metabolomics and lipidomics are shown in Figure 1.

## 3. Application of Omics in the Study of Economic Traits of Livestock and Poultry

The combined application of metabolomics and lipidomics provides an unprecedented systems biology perspective for elucidating and improving the economic traits of livestock and poultry. This integrated strategy can simultaneously capture comprehensive information on small-molecule metabolites reflecting the physiological state of the organism and lipids that constitute cellular structures and serve as signaling molecules, thereby accurately revealing the intrinsic molecular regulatory networks that influence the formation of meat and egg yield, flavor, and nutritional quality [60,61,62,63,64]. By identifying key metabolite and lipid biomarkers that are strongly associated with important economic traits, this approach can guide precision nutritional intervention, enabling whole-process optimization of livestock and poultry economic traits from genetics to management, and ultimately driving the high-quality development of meat and egg yield and quality.

### 3.1. Application of Metabolomics and Lipidomics in Meat Quality

Widely applied complementary approaches characterize biochemical variations associated with quality traits. Key attributes such as flavor, tenderness, and juiciness are closely associated with metabolites and lipid composition, reflecting both metabolic activity and lipid deposition processes [66,67,68]. More importantly, rather than acting independently, metabolites and lipids interact dynamically within metabolic networks, jointly shaping meat quality traits across different species and production systems. The integration of metabolomics and lipidomics not only enables comprehensive detection of small molecules but also facilitates the construction of global metabolic profiles. Recent multi-omics studies have further demonstrated that coordinated changes in metabolic pathways and lipid signaling are critical determinants of meat quality variation, particularly across breeds and muscle types [69]. However, despite the growing number of studies, differences in experimental design, animal species, and analytical platforms often lead to inconsistent findings, highlighting the need for comparative synthesis and standardized methodologies.

Accumulating evidence suggests that amino acids and nucleotides are primary contributors to meat flavor across multiple livestock species. For example, inosine monophosphate (IMP) consistently contributes to umami taste in chicken, duck, pork, and beef, while glutamic acid and aspartic acid enhance flavor intensity [70,71,72,73]. Notably, while these metabolites are widely conserved flavor contributors across species, their relative abundance and contribution vary depending on genetic background, growth rate, and feeding strategies, indicating species-specific metabolic regulation mechanisms. In addition to metabolites, lipid molecules play a dual role in meat quality by acting both as flavor precursors and as structural components influencing intramuscular fat (IMF) deposition [74]. Recent integrative metabolomics and lipidomics analyses have revealed that IMF accumulation is regulated by coordinated shifts in amino acid metabolism and glycerophospholipid metabolism, providing a mechanistic link between metabolic activity and lipid deposition [65,75]. Across species, consistent patterns have been observed. For instance: In sheep, higher IMF levels are associated with increased triglycerides (TG) and diacylglycerols (DG), contributing to improved flavor [76]. In pigs, high-IMF muscle shows elevated phospholipids such as phosphatidylinositol and phosphatidylserine [77]. In poultry, age-dependent increases in glyceride accumulation lead to enhanced IMF deposition [78,79]. These findings collectively suggest that, despite species-specific differences in lipid composition, triglycerides and phospholipids are consistently associated with improved meat quality traits. Furthermore, cholesterol metabolism has also been implicated in regulating IMF deposition and peripheral fat accumulation, as observed in Tan sheep, where enhanced cholesterol homeostasis signaling contributes to improved meat quality [80].

Taken together, current studies demonstrate that meat quality is governed by the coordinated regulation of metabolite composition and lipid metabolism rather than by individual biomarkers alone [69,81]. Amino acids and nucleotides primarily influence flavor formation, whereas lipid molecules contribute through both flavor precursor generation and regulation of fat deposition. Importantly, the interaction between these two molecular classes represents a key mechanism underlying meat quality differences across species [81,82]. However, most existing studies focus primarily on a single species or experimental system, which limits the comparability of findings across different studies. There remains a lack of systematic integration of research findings, particularly with regard to distinguishing between universally conserved biomarkers and species-specific features. The combined application of metabolomics and lipidomics provides a powerful framework for addressing this limitation by enabling the identification of interacting biomarkers and the construction of regulatory networks governing meat quality [65]. As summarized in Table 1, although different livestock species exhibit distinct biomarker profiles, several common patterns can be identified, including the central role of glutamic acid in flavor formation and triglycerides in IMF-related quality traits. This highlights both the conservation and diversity of molecular mechanisms underlying meat quality.

Table 1 summarizes representative biomarkers across different livestock and poultry species, revealing that certain conserved biomarkers, such asglutamate and IMP, are consistently identified as key factors in umami formation across multiple species. Meanwhile, triglycerides and phospholipids have repeatedly been shown to be associated with IMF deposition and meat quality improvement. Certain biomarkers, such as specific phospholipids or metabolic intermediates, exhibit differences across species, reflecting variations in metabolic pathways and physiological characteristics. These findings suggest that future research should focus on distinguishing between universal biomarkers and species-specific biomarkers.

### 3.2. Application of Metabolomics and Lipidomics in Egg Quality

Egg quality represents a key economic trait in livestock and poultry production, encompassing nutritional value, flavor, and functional properties. The integration of metabolomics and lipidomics has emerged as a powerful strategy to systematically characterize the molecular basis of these traits. Rather than merely quantifying individual metabolites, combined omics approaches enable the reconstruction of metabolite–lipid interaction networks, thereby providing a more comprehensive understanding of the biochemical mechanisms underlying egg quality.

Amino acid metabolism plays a central role in determining the nutritional and sensory properties of eggs across different species. For example, glutamic acid consistently contributes to the umami flavor of eggs, while essential amino acids such as leucine are critical for nutritional quality [89,90,91]. However, their role is not limited to directly influencing compositional profiles. Evidence from comparative studies suggests that amino acids and their precursors can indirectly regulate egg quality by influencing host physiology, such as through interactions with the gut microbiota [92,93]. This highlights a conserved mechanism across poultry species whereby metabolite-mediated host–microbiota interactions lead to differences in egg quality.

Lipid composition, particularly that of the egg yolk, is a key factor determining flavor and texture. The application of metabolomics and lipidomics in egg quality research has gradually expanded from descriptive analyses of a single poultry species to comparative studies involving multiple poultry species. Numerous studies have employed non-targeted metabolomics, lipidomics, and volatile flavoromics techniques to systematically analyze whole eggs, egg yolks, or egg yolk granules from various poultry species, including pigeons, chickens, ducks, geese, quails, turkeys, and partridges [94]. These studies consistently indicate that the major differential metabolites in eggs from different poultry species are primarily concentrated in categories such as nucleotides and their derivatives, amino acids and their metabolites, and organic acids. Meanwhile, differential lipids are primarily enriched in glycerophospholipids (such as PC and PE), glycerides (TAG and DG), and sphingolipids. Among these, unsaturated fatty acids and their derived phospholipids (such as PE and PC) and glycerides have been identified as key precursors of major flavor compounds [95,96]. The exceptional flavor characteristics exhibited by certain indigenous breeds are associated with their unique lipid metabolism profiles; for example, the mushroom-like aroma of BIAN chicken eggs suggests that genetic background shapes egg quality by regulating lipid metabolic pathways [97]. This cross-system comparison highlights the importance of integrating lipidomic characteristics with genetic and physiological contexts.

Overall, the combined application of metabolomics and lipidomics has shifted egg protein research from descriptive compositional analysis to a systematic understanding. By integrating findings across different species, breeds, and experimental conditions, these methods have revealed metabolic pathways that regulate egg proteins, which are both conserved and diverse. This framework provides a more solid foundation for improving egg protein quality through nutritional and genetic interventions.

### 3.3. The Impact of Nutritional Regulation on Meat Quality

Meat quality is shaped not only by the endogenous metabolic and lipid composition of muscle, but also by nutritional interventions that alter these molecular profiles before slaughter. In this context, the combined application of metabolomics and lipidomics provides more than a descriptive inventory of compounds. It helps explain how dietary additives reshape amino acid metabolism, lipid deposition, redox balance, and energy utilization, and how these molecular shifts are translated into measurable changes in meat color, water-holding capacity, tenderness, oxidative stability, and flavor [81,98]. Therefore, omics-based studies are particularly valuable for linking nutritional strategies with the biological pathways that underlie meat quality improvement.

Across livestock and poultry species, different classes of nutritional additives appear to converge on several major biological processes. First, flavor-related traits are closely associated with changes in amino acid and nucleotide metabolism. Supplementation with specific amino acids can modify the relative abundance of taste-active amino acids in muscle, thereby affecting sweetness and umami perception in meat [99,100]. In parallel, some plant-derived additives can increase flavor-related metabolites such as inosine monophosphate (IMP), suggesting that nutritional regulation may improve flavor not only through protein composition, but also through postmortem flavor precursor accumulation [101,102]. Second, intramuscular fat deposition is strongly influenced by dietary regulation of lipid metabolism. Fatty acid supplementation and other energy-related additives can promote intramuscular fat accumulation and alter fatty acid composition, which in turn affects juiciness, tenderness, and nutritional value [103,104,105]. These findings indicate that lipid remodeling is one of the central mechanisms by which nutrition improves meat quality.

Meat lipids, especially unsaturated lipids, are highly prone to oxidation after slaughter, which accelerates quality deterioration. Several studies indicate that antioxidant additives or plant-derived extracts enhance antioxidant capacity by increasing antioxidant metabolites or enzyme activities, including total superoxide dismutase, thereby slowing lipid peroxidation and helping preserve color stability and sensory quality [106,107]. At the same time, improvements in tenderness and water-holding capacity are frequently associated with shifts in membrane lipid composition, protein degradation, and postmortem energy metabolism, rather than with a single isolated metabolite [101,108]. These findings strengthen the omics-based interpretation that tenderness, color stability, and water retention are coordinated outcomes of broader metabolic remodeling.

Comparative evidence also suggests that the phenotypic emphasis of nutritional regulation differs among species. In broilers, dietary additives more often improve oxidative stability, drip loss, cooking loss, and flavor-related metabolites, which may reflect the rapid growth rate and distinct muscle characteristics of poultry [102,109,110]. In pigs, additives such as succinate and calcium are more frequently linked to intramuscular fat deposition, reduced shear force, and improved color traits [111,112]. In ruminants, guanidinoacetic acid has shown beneficial effects on pH, antioxidant status, water-holding capacity, and meat color in both sheep and cattle [102,113], suggesting that some metabolic targets are conserved across species despite differences in digestive physiology. Taken together, these comparisons show that nutritional intervention has shared mechanistic themes, but species-specific responses remain important when translating omics evidence into practical feeding strategies.

Therefore, the main contribution of integrated metabolomics and lipidomics is not only to identify responsive metabolites or lipid classes, but also to reveal how these changes are organized into pathways linked to final meat-quality phenotypes. The most recurrent pathways include amino acid metabolism, lipid metabolism, energy metabolism, and antioxidant defense, and these pathways collectively influence flavor, tenderness, water-holding capacity, color stability, and nutritional composition. As shown in Table 2, although probiotics, functional lipids, metabolic modulators, minerals, organic acids, and plant extracts vary in form, they ultimately influence a limited set of core traits, particularly oxidative stability, fat deposition, color, and tenderness. Precision nutrition should focus on coordinated metabolic regulation rather than isolating individual quality indicators.

Overall, Table 2 shows that although the additives differ substantially in chemical nature and application strategy, their reported effects converge on a small number of recurrent biological outcomes. Most interventions improve meat quality through one or more of four routes: enhancing antioxidant protection, promoting favorable lipid remodeling, stabilizing postmortem muscle metabolism, or improving flavor-related metabolite profiles. This pattern reinforces the need to interpret nutritional regulation through integrated omics evidence rather than through isolated production traits alone.

### 3.4. The Impact of Nutritional Regulation on Egg Quality

Egg quality is influenced by dietary additives not simply through the direct deposition of nutrients into eggs, but through broader metabolic remodeling in laying hens. Compared with meat, egg formation is a continuous biological process that depends on the digestion, absorption, transport, and selective deposition of nutrients into the yolk and albumen. For this reason, the combined use of metabolomics and lipidomics is particularly valuable in egg-quality research, because it can distinguish direct enrichment effects from indirect physiological regulation, including changes in intestinal absorption, hepatic lipid metabolism, antioxidant status, and yolk precursor transport. This integrated approach therefore provides mechanistic insight into how dietary additives alter egg color, flavor, antioxidant capacity, and nutrient composition, rather than merely documenting changes in conventional egg-quality indices.

Micronutrient fortification and antioxidant protection. Selenium-related additives (including selenium-fortified yeast and hydroxyselenomethionine) can consistently increase selenium deposition in eggs and enhance antioxidant capacity [116,117,118,119]. Similar antioxidant-promoting effects have also been reported for plant-derived additives such as *Cardamine hupingshanensis*, suggesting that additives with different chemical properties may converge on a shared redox-regulatory mechanism [120]. From an omics perspective, these responses are important because antioxidant improvement is not only reflected in a higher concentration of functional compounds in eggs, but also in changes in oxidation-related metabolites and lipid stability. This is one reason why integrated metabolomics and lipidomics provides stronger mechanistic evidence than a single-indicator evaluation. Egg yolk color is determined not only by the amount of pigment supplied in the diet, but also by the efficiency of absorption, transport, and deposition into the yolk. Studies have shown that reducing dietary vitamin A can enhance carotenoid deposition efficiency, while additives such as curcumin, Urtica dioica, black soldier fly larvae, and several algal resources also improve yolk color or increase carotenoid-related indices [121,122,123,124,125]. These findings suggest that different nutritional strategies may improve yolk pigmentation through partially overlapping pathways involving carotenoid availability, lipid-mediated transport, and antioxidant stabilization.

Coordinated changes in amino acid and lipid metabolism can modulate the flavor and nutritional composition of eggs. Insect-derived ingredients and oil-based additives can alter the amino acid balance, fatty acid composition, phospholipid profile, cholesterol content, and choline levels in eggs [126,127,128,129]. These changes are particularly important because the flavor and nutritional value of eggs are determined jointly by water-soluble metabolites and fat-soluble compounds. In this context, metabolomics can detect changes in flavor-related amino acids and other small molecules, while lipidomics can capture changes in phospholipids, monounsaturated fatty acids, and polyunsaturated fatty acids that influence yolk texture, flavor stability, and nutritional quality. Therefore, integrating these two omics approaches is crucial for understanding how functional additives alter egg quality at the molecular level.

As shown in Table 3, although the sources of additives tested in laying hens varied significantly—including plant extracts, enzyme preparations, selenium sources, oils, insect-derived materials, and microalgae—their effects were primarily focused on enhancing antioxidant capacity, improving yolk color, and enriching nutritional content. This suggests that different experimental systems may exert their effects through distinct mechanisms, but ultimately influence overlapping biochemical pathways. Selenium-related additives are primarily associated with mineral enrichment and antioxidant protection [118,119], while pigment-rich plant and algal additives are more closely linked to improvements in egg yolk color [120,123,124,125], and lipid-rich supplements more directly reshape the lipid composition and nutritional profile of the egg yolk [129].

**Figure 2:**
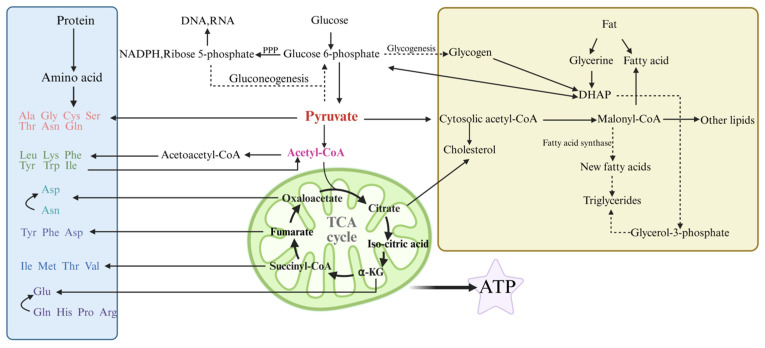
Shows the metabolic cycle diagram of the three major nutrients.

As shown in Figure 2, nutritional regulation strategies directly participate in the metabolic processes of nutrients in the body of livestock and poultry by adjusting feed components. After digestion and absorption, exogenous nutrients are integrated into the metabolic networks of the three major nutrients: proteins, lipids, and carbohydrates. These three are interconnected and transformed through intermediate metabolites, together forming the biochemical basis for the formation of meat and egg quality.

## 4. Multi-Omics Integration and the Application of Artificial Intelligence

The integration of multi-omics and artificial intelligence is profoundly transforming the research paradigm of economic traits in livestock and poultry. This strategy integrates vast amounts of biological data and employs AI algorithms to identify key regulatory networks and biomarkers that influence meat and egg quality, as well as their nutritional value and flavor. This approach not only significantly overcomes the limitations of single-omics analysis in terms of data dimensions and information depth but also effectively addresses the technical challenges posed by traditional statistical methods in handling high-dimensional, nonlinear, and multifactorial interactions, thereby providing a solid foundation for improving meat and egg quality.

### 4.1. Integration of Cross-Omics Data to Analyze the Association Between Genetics and Phenotypes

Multi-omics technologies can effectively overcome the limitations of single-omics analysis, thereby systematically elucidating the complex regulatory networks involved in the formation of meat and egg quality. By integrating multi-dimensional data such as genomics, transcriptomics, proteomics and metabolomics, multi-omics methods construct a full-chain regulatory pathway from upstream genetic variation, midstream transcriptional and translational regulation to downstream metabolic phenotypes, revealing the molecular network basis for the formation of meat quality and egg quality.

In existing studies, integrated multi-omics analyses have successfully identified some biomarkers and regulatory mechanisms affecting meat and egg quality, providing important clues for understanding the molecular basis of these complex traits. For example, in cattle, integrated multi-omics analysis revealed that the TGF-β signaling pathway serves as a key regulatory axis in the differential distribution of linoleic acid among different breeds [131]. Meanwhile, IMP, GSH, and EGT have been identified as key metabolic markers affecting flavor and color stability, with IMP consistently recognized as a major contributor to umami taste across multiple species [132]. In pigs, integrated transcriptomic and lipidomic analyses revealed the synergistic roles of genes such as *ACC1* and *FASN*: they promote the synthesis of palmitic acid, which is subsequently elongated and desaturated by *ELOVL6* and *SCD* to form long-chain fatty acids required for triglyceride (TG) synthesis; *DGAT2* catalyzes extensive TG synthesis, and under the regulation of *PLIN1*, TG is stored in lipid droplets [133]. The efficient operation of this pathway is considered a key factor contributing to high IMF content. Additionally, 14 volatile organic compounds (VOCs, e.g., pyridine, 1-hexanol, phenol), 15 differentially expressed genes (DEGs, e.g., *ACAA2*, *HADHB*, *CPT1B*), and 10 differentially expressed lipids (DELs, e.g., PC(18:1e_6:0), PC(26:3), PE(34:6e)) have been identified as potential contributors to pork flavor [134]. Cross-species comparisons indicate that glycerophospholipids and triglycerides are important lipid precursors for meat flavor formation [135,136,137], while the PI3K-Akt, MAPK, AMPK, and PPAR signaling pathways are commonly enriched in meat quality regulation across different species [138,139,140,141]. In egg quality research, transcriptomic and proteomic analyses revealed that KRT14 may affect eggshell structure and quality by regulating calcium metabolism and calcium carbonate deposition in the eggshell [142]. In goose eggs, integrated lipidomic, metabolomic, and proteomic analyses revealed differences in quality and nutrition among different breeds. Both differentially expressed proteins (DEPs) and differentially expressed metabolites (DMs) were enriched in the vitamin digestion and absorption pathway as well as the neuroactive ligand–receptor interaction pathway, suggesting that these two pathways may serve as core regulatory hubs linking nutrient metabolism to egg quality traits [143].

However, these findings also expose several common limitations and challenges in current integrated multi-omics research. First, most studies remain at the level of “associative description”; they identify genes, metabolites, or lipids associated with phenotypes through differential analysis and infer functions based on pathway enrichment results, but lack causal validation. For example, the role of the TGF-β pathway in linoleic acid metabolism in cattle has not been directly validated by experimental approaches such as gene knockout, overexpression, or pharmacological intervention [131]. Similarly, although the gene co-network associated with IMF deposition in pigs (*ACC1*-*FASN*-*ELOVL6*-*SCD*-*DGAT2*-*PLIN1*) is logically coherent, the actual in vivo regulatory relationships and quantitative contributions remain to be confirmed by functional experiments [133]. Second, most studies simply overlap or correlate differential lists from different omics layers and construct simple co-expression networks, but rarely employ systems biology methods to distinguish “correlation” from “causation”, and lack dynamic modeling across tissues or time dimensions.

### 4.2. Artificial Intelligence Empowers Omics Data Analysis and Decision-Making

Artificial intelligence technologies are deeply empowering the analysis and decision-making processes of omics data. Faced with the massive and high-dimensional data generated by multi-omics integration, artificial intelligence algorithms represented by machine learning and deep learning can efficiently mine the hidden complex patterns and nonlinear relationships, and systematically elucidate the multi-level regulatory networks from genomic sequences to phenotype formation.

In meat and egg quality research, analytical frameworks based on artificial intelligence (AI) are gradually forming a standardized paradigm for multi-omics data analysis, significantly improving the processing efficiency of high-dimensional data and the robustness of biomarker discovery. The integration of AI into multi-omics data analysis has established a complete analytical pipeline covering data exploration, quantification of environmental factors, feature selection, and classification prediction. Principal component analysis (PCA) is suitable for exploratory visualization and outlier detection; redundancy analysis (RDA) can quantify the statistical significance of environmental factors on metabolic profile variation; and the combined use of partial least squares (PLS) and random forest (RF) can effectively handle collinearity issues in high-dimensional data while capturing complex non-linear relationships, thereby enabling comprehensive dissection of flavor marker compounds and cross-sample classification prediction [144].

Taking egg quality research as an example, researchers first employed the K-nearest neighbors algorithm for missing value imputation and normalization of volatile metabolomics and lipidomics data. Subsequently, five supervised learning models—Gaussian Naïve Bayes, logistic regression, random forest, support vector machine (SVM), and XGBoost—were constructed to classify and discriminate five types of poultry eggs. Using recursive feature elimination with cross-validation (RFECV), with logistic regression and random forest as base models, redundant features were iteratively removed, refining hundreds of candidate features identified by conventional differential analysis into a set of nine core biomarkers that intersected between the two models. Finally, the selected biomarkers were combined with Spearman correlation analysis to construct a transformation network linking lipid precursors to key flavor compounds, endowing the AI-screened results with clear biological interpretability [145]. This paradigm transforms multi-omics feature selection into a machine-learning-driven problem of prediction and iterative elimination, significantly improving the efficiency and robustness of biomarker discovery.

Similar methodological systems have been successfully applied in meat quality research. For example, non-targeted metabolomics combined with random forest algorithms has been used to analyze dynamic metabolic changes in goose meat during long-term storage [146]. In lamb origin traceability studies, random forest recursive feature elimination (RF-RFE) initially identified 29 potential biomarkers from high-dimensional metabolomics data; these biomarkers exhibited significant breed-specific and production-environment-related variations. When further combined with the Naïve Bayes algorithm, they achieved the highest classification accuracy among all evaluated machine learning methods. Compared with traditional univariate or simple multivariate statistics, RF-RFE provides a more practical feature selection approach for handling high-dimensional metabolomics datasets [147]. It is worth noting that the analytical framework described above is applicable not only to the dissection of flavor compounds but can also be extended to predictive modeling of key meat quality indicators such as tenderness, juiciness, water-holding capacity, and color stability.

Although the integration of AI into multi-omics data analysis has demonstrated strong capabilities in biomarker screening and classification prediction, several challenges remain to be addressed. For instance, missing value imputation methods in data preprocessing (e.g., K-nearest neighbors) may introduce bias due to assumptions about the missing data pattern. While cross-validation helps control overfitting, most studies lack independent external validation. Although the feature importance ranking from ensemble models such as random forest enhances interpretability, deep learning models still face a “black box” dilemma. Moreover, non-biological variations such as batch effects and instrument drift, if not adequately corrected, may be erroneously learned by models as “biomarkers.” Therefore, future research should focus on standardized preprocessing workflows, external validation, integration of causal inference algorithms, and functional validation experiments, so that AI-driven multi-omics analysis can truly move from “predictive association” to “verifiable mechanistic dissection”.

## 5. Conclusions and Future Perspectives

This review shows that the value of metabolomics and lipidomics in livestock and poultry research lies not simply in detecting large numbers of small molecules, but in identifying recurrent molecular patterns that are consistently associated with meat and egg quality traits. Across the studies reviewed here, several biomarkers emerge as relatively robust indicators. In meat, glutamic acid, aspartic acid, and inosine monophosphate (IMP) are repeatedly linked to umami intensity and flavor formation, whereas triglycerides, diacylglycerols, and major phospholipid classes such as phosphatidylcholine and phosphatidylethanolamine are consistently associated with intramuscular fat deposition, tenderness, and flavor precursors. In eggs, glutamic acid and leucine are among the most recurrent metabolite markers related to nutritional and sensory quality, while yolk glycerides and glycerophospholipids, particularly PC- and PE-related lipids, are repeatedly implicated in flavor, texture, and nutritional value. In addition, some metabolite groups, including biogenic amines, oxidized fatty acids, taurine, cholic acid, butyric acid, and histamine, also show promise in traceability, freshness evaluation, and disease diagnosis.

At the same time, review literature indicates that no single biomarker is sufficient to explain the complex quality traits observed across different species or production systems. Most traits are shaped by synergistic changes in amino acid metabolism, lipid remodeling, energy metabolism, and antioxidant pathways. Therefore, metabolomics and lipidomics offer the greatest informational value when used as integrative tools to define biomarker combinations and pathway-level signatures, rather than merely for identifying isolated candidate compounds. However, several methodological challenges continue to limit cross-study comparability and biological interpretation. These challenges include differences in species, breeds, tissues, feeding systems, and sampling designs; inconsistencies in extraction procedures, analytical platforms, and data processing workflows; limited reliability of metabolite and lipid annotation in non-targeted studies; insufficient correction for batch effects and instrument drift; and the frequent lack of independent external validation. In multi-omics and AI-assisted studies, another major limitation is that many reported associations remain merely correlations, lacking sufficient validation of causality through functional experiments.

Future work should therefore move in three directions. First, the field needs more standardized workflows for sample collection, QC design, annotation, and reporting, so that biomarker reproducibility can be evaluated across laboratories and species. Second, future studies should prioritize longitudinal, tissue-specific, and multi-omics designs to distinguish universal biomarkers from species-specific signals and to better resolve gene–environment–microbe interactions underlying quality traits. Third, AI and machine-learning approaches should be combined with external validation, causal inference, and experimental verification to move biomarker discovery from predictive association toward mechanistic interpretation and practical application. With these advances, metabolomics and lipidomics are expected to provide a stronger foundation for precision nutrition, quality assessment, disease monitoring, and genetic improvement in smart animal production.

## Figures and Tables

**Table 1:** Biomarkers in Meat Quality.

Animal Species	Sample	Biomarker	Function	References
Cattle	longissimus	Glutamic acid (Glu)	Enhance umami	[83]
	Leg muscle, Flank muscle	PE, SM, CAR	Improve tenderness	[84]
Pig	longissimus dorsi muscle	L-Malic Acid, β-Alanine (Acpc)	Improve meat quality	[85]
Sheep	biceps femoris	IMP, DAG 36:3, TAG 40:0 and 46 other types	Change the flavor	[86]
Broiler	thoracic muscle	TG, FA, CE	Enhance flavor	[65]
Goose	thoracic muscle	Cysteine (Cys) and Glucose-6-phosphate (G6PD)	Enhance flavor	[87]
Camel	longissimus dorsi, psoas major muscle, semitendinosus muscle	Phosphoenolpyruvate (PEP), L-phenylalanine, PC	Increase IMF, enhance tenderness	[88]

**Table 2:** Effects of Different Nutritional Additives on Meat Quality.

Nutritional Additives	Animal Species	Function	References
Bacillus subtilis 7.0 × 10^7^ CFU/g, Bacillus licheniformis 4.1 × 10^7^ CFU/g	Broiler	Reduce cooking losses	[109]
Glycerol Monolaurate (GML)	Broiler	Reduce lipid peroxidation rate, increase the USFA/SFA ratio, and improve tenderness	[110]
Guanidinoacetic acid (GAA)	Sheep	Increase pH levels, enhance water-holding capacity and total antioxidant capacity, and reduce protein degradation	[114]
	Cattle	pH value increases, improving L* and a* values (L* decreases, a* increases), significantly reducing drip loss and cooking loss, and enhancing water-holding capacity	[113]
succinate	Pig	Increase intramuscular fat content, reduce shear force and cross-sectional area	[111]
Calcium	Pig	Significantly improve the color of the longest back muscle, reduce backfat thickness, and increase intramuscular fat content	[112]
Ampelopsis grossedentata Extract (AGE)	Broiler	Reduce muscle shear force and drip loss, increase the a* value, and significantly boost inosine monophosphate (IMP) levels	[101]
Dried cherry pomace	Broiler	Reduce drip loss and lower crude fat content	[115]

**Table 3:** Effects of Different Nutritional Regulations on Egg Quality.

Nutritional Additives	Animal Species	Function	References
*Cardamine hupingshanensis* (CDH)	Layer	Improve yolk color, egg shape index, increase shell thickness, and enhance antioxidant capacity.	[120]
*Urtica dioica* L. (SN)	Layer	Increase carotenoid, a*, and b* values	[123]
Protease DE200	Layer	Reduce egg yolk moisture content and increase Haugh units	[130]
Selenium-Enriched Yeast (SY)	Layer	Increase selenium concentration to enhance antioxidant capacity	[118]
soybean oil	Layer	Increase the content of monounsaturated fatty acids, cholesterol, phospholipids, and choline, and increase the content of essential and non-essential amino acids	[129]
Hydroxy selenium methionine (OH-SeMet)	Layer	Enhance antioxidant capacity and increase selenium content	[119]
Black soldier fly larvae (BSF)	Layer	Improve egg yolk color	[124]
*Trachydiscus minutus*, *Japonochytrium marinum*, *Scenedesmus obliquus*, *Chlorella vulgaris* and *Vischeria helvetica*	Layer	Increase the concentration of organic selenium, polyunsaturated fatty acids, and carotenoids	[125]

## Data Availability

No new data were created or analyzed in this study. Data sharing is not applicable to this article.
